# Supplementary material for: Hypermethylation at 45S rDNA promoter in cancers
Source: PLoS One. 2025 Jan 7;20(1):e0311085. doi: 10.1371/journal.pone.0311085 (PMC11706406; doi:10.1371/journal.pone.0311085)
Supplement: S1 Table — (DOCX) [file pone.0311085.s001.docx]

**Hypermethylation at 45S *rDNA* promoter in cancers**

Trang Thi Quynh Tran^1,2#^, Trang Hien Do^1#^, Tung The Pham^1^, Phương Thi Thu Luu^1^, Oanh Minh Pham^1^, Uyen Quynh Nguyen^2^, Linh Dieu Vuong^3^, Quang Ngoc Nguyen^3^, Tuan Van Mai^3^, Son Van Ho^4^, Than Thi Nguyen^4^, Lan Thi Thuong Vo^1,2*^

**S1 Table**. Primer sets and quantitative real-time PCR conditions for measurement of *rDNA* methylation and detection of the native *rDNA* sequences. The methylation-independent-specific PCR (MIP) primers (Ref-F/R) are designed from the consensus sequences of the 5’*LINE-1* region (X58075.1) and the methylation-dependent-specific PCR (MSP) primers (rDNA-Me-F/R) are designed from a core promoter of the *rDNA* genes (U13369.1). All non-CpG cytosines in the MIP and MSP primer sets have been replaced by ‘t” in the forward primers and by “a” in the reverse ones

|  | | **Primers** | **Sequence (5’–3’)** | **Amplicon size (bp)** | **PCR conditions** |
| --- | --- | --- | --- | --- | --- |
| Quantitative measurement of *rDNA* methylation | MIP primers | Ref-F | tAtAGtAGTtTGAGATtAAAtTGtAAGG | 84 | 95°C 2 min, 40 cycles of (95°C 15 sec, 61°C 1 min) |
|  |  | Ref-R | aTTTACCTAAaCAAaCCTaaaCAATaaC |  |  |
|  | MSP primers | rDNA-Me-F | GGATAGGTGTtCGTGTCGC | 70 |  |
|  |  | rDNA-Me-R | CCCGaaaCCGaaAaaTCGCG |  |  |
| Detection of the native *rDNA* sequences (PCR) | | rDNA-Native-F | GCTCCCGCGTGTGTCCT | 221 |  |
|  |  | rDNA-Native-R | CAGGTCGCCAGAGGACAG |  |  |
